# Unmasking State Harm: The Border as a Theatrical Space of Gendered Violence

**DOI:** 10.1177/10778012231162037

**Published:** 2023-03-14

**Authors:** Marinella Marmo

**Affiliations:** 1065 Flinders University, Bedford Park, SA, Australia

**Keywords:** state harm, gendered and racialized border violence, border performativity, border cavity searches

## Abstract

Systemic gendered violence at the borders of the Global North is perpetrated by state actors and their proxies, and yet remains underdiscussed. This article presents numerous cases of such violence to argue that it is not random, does not occur in a vacuum or at the whim of wayward officers. Rather, this gendered state violence is the expression of a patriarchal and hegemonic state that positions the woman at the border as out of place. Deploying performative theories, this article unmasks the masculine agenda enacted at the border to reveal that its violent exhibition of power serves many purposes.

## Prologue

Having been raised in Italy, I could not have missed the monologue entitled “*The Rape*” performed by the actress and political activist, Franca Rame. “*The Rape*” was a theatrical representation of Rame's own experience of abduction, rape, and torture that took place on March 9, 1973 (Rame, 1975). I was a naive teenager when she performed it live nationally in 1988 on the most-watched TV show of the time (*Fantastico*) on the biggest state-sponsored channel, RAI UNO. It was probably the first time I had been exposed to Rame's work. It was difficult to make sense of what I was witnessing on primetime television: one minute there was dancing and laughing, the next an account of a rape, with the recounting itself an act of empowerment and political resistance, as Luciana d’Arcangeli has argued (2009).

In “*The Rape*,” as she stands outside the police station, Rame also shares her internal dialogue about the reasons why she did not report the violence to the police: “I think of what I would have to face if I went in to [the police station]. I can hear their questions. I can see their faces … I can see them laughing … […] I’ll report them [the perpetrators] tomorrow.”

But there is more to this story. What I did not know then, and what took 25 years from the original event to become public knowledge in Italy, was that the news of the rape was welcomed with applause and cheers by the Pastrengo (a municipality in the province of Verona) division of the Italian *carabinieri* police. Further, while no trial took place, numerous substantiated claims have been made by Italian prosecutors and other sources that top Italian political leaders mandated the criminal act of rape (Tiso, 2013), which was then authorized by the *carabinieri* (Osservatorio, 2013). Thus, I argue, this was a case of rape by the state; Rame was punished and silenced by the masculine state.

Franca Rame reclaimed power as an act of resistance by representing the truth of what happened to her as a theatrical monologue (d’Arcangeli, 2009). Yet this episode, for which there has been no closure or “justice” (juridical or otherwise), has left Italian women feeling anguish over questions that have not gone away.^1^

Rame's theatrical piece on personal and state violence is pivotal to the key questions guiding this article. Does the state, directly or indirectly, violate, abuse, humiliate, and torture women? Does the state “laugh” when women are abused? Do these harms contradict and counteract, even now, the proclaimed progress of the state's efforts against violence against women? Initially unaware of the possible connections between gender crimes and state violence, I have been researching the gendered and racialized border for a number of years now, only to find unsatisfactory answers to all these questions.

## Gendered Violence and the Theater of the Border

This article addresses the actualizing and ritualizing—as in a theatrical performance—of gendered and racialized violence at the border, through state agents and their proxies. As many scholars note, in these borderlands women are more likely to be subjected to gendered violence (Caluya, 2019; Gerard, 2014). I argue that the nation-states of the Global North are co-perpetrators of such violence.

Gendered border violence as a normalized state practice (Marmo, 2019; Smith & Marmo, 2014) may appear to contradict internal government policies on violence against women. However, in reality, such violence at the border is part of a continuum of gendered violence. Everyday gendered violence is related to the multi-faceted politics of disempowerment of women, where the public and private spheres interact to enforce this disempowerment, yet this remains hidden by state policies on domestic and family violence. However, it is at the border where the contradictory state narratives on women's rights become clear. The state and the border narratives of gender disempowerment are intertwined, yet it is only at the border that the state's real patriarchal and hegemonic intention is unmasked (Mountz & Hyndman, 2006).

Drawing on this notion of the masculine state and its agenda of dominance (Brown, 1992; Young, 2003), this article focuses on how gendered border practices clearly illuminate the “spaces” where state can trespass on the woman's body. This allows the state to advance its masculine agenda, justified on the basis of security imperatives that minimize or avoid state responsibility and accountability. Embedded within this logic is the patriarchal view of male rights and privileges, enabled by the state of exception at the border (Agamben, 2005). This article approaches the topic by arguing that the border can be understood as a theatrical site where the performance of gendered violence is necessary to imprint masculine state powers on women's bodies. The border thus requires a performance to send a message to an audience (Goffman, 1959) that includes everyone: those subjected to the violence, other travelers, and the public.

Scholars and practitioners within the arts have been exploring the liminal space of the state border and its impact on people, experimenting with different typologies and techniques (Cox, 2015; Cox & Wake, 2018; Guterman, 2014; Jeffers, 2012; Noriega, 2016). Artists and activists are capable of pushing and lifting the border *boundaries* imposed by a narrative of state security, to allow the acts that occur at the border to reach our gaze, thereby improving our understanding of this liminal space. A few scholars have analyzed the border as a theatrical space (Amoore & Hall, 2010; Nield, 2008; Salter, 2007). Like Mountz and Hyndman (2006), I argue that the border is a theater in which state violence is enacted with impunity. Dario Fo, a celebrated artist and thinker, as well as the life partner of Franca Rame, talked about his preference for a “theater of situation” (Hirst, 1989, pp. 37–38), as opposed to a “theater of characters,” because the situation allows for the unfolding of the plot, so the action is pivotal. In this vein, the characters of immigration officers, state police, and other state proxies can be seen as interchangeable, as it is the space of the border that is conducive to power and gendered inequality, which allows the plot to unfold.

This article is divided into three main parts. In the first part, “Theatrical Productions of Selective Border Violence,” a short background discussion sets the scene for the border as a theatrical space, with its performance of gendered violence designed and actualized by the state for the purpose of advancing its hegemonic and patriarchal agenda. The second part, “Gendered-Racialized Borders,” explains the gendered-racialized border as a space where “truth” is fabricated to fit the narrative of security through the “theater of paperwork” and the “theater of the body.” The theatrical performance is discussed as essential to produce sovereignty and to assign identities of inclusion/exclusion. Through such performance, the state can “show its doing,” and project its prevailing message of security to its audiences, from actual travelers to the wider public back home. The third section, “Disciplining the Gendered Body—Inscribing State Power onto the Body,” discusses case studies of border violence to showcase the ways in which state power is inscribed on the gendered body. These examples are selected to illustrate the ubiquitous and systemic nature of the theater of situation at the Global North borders and to demonstrate how gendered border violence perpetrated by the patriarchal state occurs “on the ground.” The conclusion returns to reflect on Franca Rame's “*The Rape*” and the meaning of the state's judgment of women as “out of place” at the border.

## Theatrical Productions of Selective Border Violence

Diverse artistic representations and performances of border crossing and its associated violent elements have been explored for some years, and many academic reflections on such productions are now available (including, among others, Cox, 2015; Cox & Wake, 2018; Guterman, 2014; Jeffers, 2012; Lanz, 2016; Noriega, 2016). As pointed out by Staudt (2008, p. 18), the purpose of such representations is to raise awareness: “[p]erformance is a useful concept around which to analyze anti-violence social movements at the border.” In Italy, for example, every year there are hundreds of these representations (such as Teatro di Roma, 2019) using different means, which are produced and acted, in some cases, by those subjected to border practices (see, e.g., Cimoli, 2014).

In many such artistic productions, site and performance become intertwined; the site brings the performance alive, augmenting its aesthetic, cultural, and political effects (Tompkins, 2012). Site-specific theater has many advantages, including that it creates coexistence between the geographical place and the performance, which enables multiple layers of interpretation: “[t]he multiple meanings and readings of performance and site intermingle, amending and compromising one another” (Pearson & Shanks, 2001, p. 23).

Some scholars have explored the border itself as a theatrical space (Amoore & Hall, 2010; Nield, 2008; Salter, 2007). Indeed, through the lens of performative theories, the border can be considered a theatrical space where the state actualizes and ritualizes selective violence to serve multiple purposes (Goffman, 1959). Borders are an ideal environment in which to define and redefine sovereignty, by selectively choosing and applying socio-cultural and legal-political elements that can reinforce state hegemony. Not only does border performance by the state produce and cement meanings, it also affects audiences. Borders have become safe spaces where the state can project and amplify an audiovisual representation of the self that no other platform can offer, an important element explored in detail later in the sub-section “As Theatrical Places of Performances (Showing/Doing).”

In such a “theatre of liminality” (Turner, 1979), the state can entrench positions of inclusion and exclusion framed by a script of security. Hence, as “otherness” is defined by sovereignty, patriarchy, and hegemony, the dominant nature of the state over the woman and her body is revealed. As discussed by Mountz and Hyndman (2006, p. 446), feminist scholarship has addressed the many ways in which global disembodied masculinity manifests as a further layer of hyper-masculinization at the border, which normalizes gendered border violence. A theatrical performance of patriarchal border power acts as a liminal phenomenon that confirms the structure of gender dominance and its various symbolisms (Turner, 1979). In this way, the state “does gender at the border” (Butler, 1995; Staudt, 2008), meaning that the state socially constructs—and maintains through the repetition of performance—the elements of gender and border as a unified agenda. And so, the everyday binaries underlying the dominant culture (man/woman, in place/out of place, white/other) are imported, applied, and amplified at the border. As Mountz and Hyndman (2006, p. 451) argue, “through dualities, borders produce and reproduce differences. They construct people as in/out, legal/illegal, here/there, white/racialized other.” Such binaries must be continually performed and reinforced to have an effect (Goffman, 1959). Borders produce and reproduce the paradoxes present in everyday domestic society in the Global North: whereby the state is seen as fighting for women's safety, equal rights, and access to justice, as per the rhetoric of fairness and equal opportunity, while producing and maintaining financial inequality, job, and health insecurity, all of which mantain a culture of fear and uncertainty.

But while within the society such contradictions are more subtle and hidden, as well as fiercely denied by those in power, at the border the woman is isolated and exposed. As Mountz and Hyndman (2006, p. 251) reflect, the border delivers “moments of truth when power that often operates more subtly is exposed in all its incarnations.” The border is therefore a multi-dimensional space where the representation of patriarchy and hegemony becomes raw and unmasked, as the filters of common sense and political correctness do not apply. Border control procedure is deeply intertwined with the control of women's bodies, and its symbolic and performative actions of denial of justice and abuse of power are predetermined not accidental. As pointed out by Goffman (1959), this is justified because the presentation of the self requires that one adapts to different social settings and adopts a different performance accordingly. Within this process, gendered violence at the border is socially and systematically constructed: first, through envisioning and enforcing a patriarchal agenda that hinders, and even renders more dangerous, women's movements (Andrijasevic, 2009; Gerard, 2014); and second, through imposing such power on the women themselves and other audiences.

Furthermore, the border is a theater of situation (as per Dario Fo's vision) because the plot of hegemony and masculinity unfolds regardless of the individual actors involved; they are interchangeable, as their role is the same in any border of the Global North. The repeated gestures of “being,” “doing,” and “showing doing” (Schechner, 2002; Turner, 1979) establish and maintain the predominance of masculine protection and security while minimizing or avoiding any close scrutiny. In this liminal space, socio-legal rules are uncertain or suspended, and outcomes are purposely ambiguous (as per Horvath et al., 2009). Yet this fluidity and randomness do not enable different possible subject positions and are not dependent on the whims of wayward immigration officers; at the border the plot proceeds as per the state's design and advances the agenda of the state (Garland, 2001). And while this agenda may appear fragmented and schizophrenic (Garland, 2001), it does constantly pull into the direction of border discrimination, based on gender and race, intersected with other relevant qualifiers, such as but not exclusively, social class, sexuality, and ideology. Indeed, the border is a legal tool to subordinate and discipline the marginalized other (Lyon, 2006; Nagra & Maurutto, 2016; Said, 1985). Viewing these selective border processes applied to the intersectional woman through the lens of performance allows us to expose their inherent violence.

## Gendered-Racialized Borders

### As Theatrical Production of Masculine Sovereignty

Borders are sites of state-making, and the constructed dominant narrative of national security and protection deliberately obfuscates any nuanced discussion of violent inclusion/exclusion based on gender, race, and other socio-cultural qualifiers. Yet, in the view of the state, gendered border violence perpetrated by state agents or their proxies is either nonexistent or peripheral to other prevailing narratives—promoted and circulated by the state—such as those around the violence inflicted by smugglers and/or third parties on female travelers (see Andrijasevic, 2009; Tessitore & Margherita, 2019; UN Women et al., 2019, among others). Undoubtedly, the intensification of state efforts to tighten borders to assert sovereignty through a narrative of securitization is directly linked to higher levels of harm experienced by transnational migrants, especially women (Aas & Bosworth, 2013; Gerard, 2014; McCulloch & Pickering, 2012; Weber & Pickering, 2011). Among these types of harm, gendered border violence perpetrated directly by the state needs to be included. Although often hidden from view and not at the center of scholarly analysis (see, e.g., Esposito et al., 2019), accounts of such violence are not uncommon, and they form a clear picture of gendered violence at the border of the Global North. Such gendered violence does not occur in a vacuum and is not tangential. Rather, it is part of an apparatus of masculine and hegemonic power exercised against women (Brown, 1992; Young, 2003), especially—though not only—through its racialized practices. The violence rests not only in doubting the veracity of women's witnessing narratives, but also in intrusive questioning techniques, which include belittling and abusive language, as well as unnecessary and invasive body checks (Marmo, 2019). Such violent encounters have been the reality within many immigration systems for decades, preceding the current regime of securitization. The case of virginity testing of fiancées from the Indian subcontinent with regular visa status at the British border is a historical testament to this (Smith & Marmo, 2014); in this case, women had to prove their virginity literally by showing that they had an intact hymen, in order to obtain permission to enter Britain with a fiancée visa. As evident in the cases discussed later, the expectation that the woman cannot be trusted and that further evidence needs to be produced, often within her body, is not just a historical phenomenon occurring at the borders of the Global North (Newsome, 2003; Yu Hsi Lee, 2015).

### As Theatrical Places of Identities: Theater of Paperwork and Theater of the Body

Border performances are highly bureaucratized, dependent on paperwork, on administrative rituals and on the cooperation of travelers. Yet, such performances embed gendered and racialized differential inclusions/exclusions that highlight the many ways some bodies at the border are seen as out of place. For some travelers, being out of place at the border becomes evident through the theater of paperwork and the theater of the body, both of which can bring about a socio-legal and juridical suspension of rights. Both inform the initial determination that a woman's story is not to be trusted by the authorities and that she needs to be detained and intrusively scrutinized. Marras (2009) recounts the comments of an Alitalia airlines security person at a busy international airport in the north of Italy, suggesting similarities to the mystery face-guessing game *Guess who*. In this regard, travelers’ characteristics are increasingly being categorized by the Italian border police, to the point that the border check has become a labeling process, as discussed in Becker's (1963)*Outsiders*. As trivial as the comparison to a children's board game may seem, its usefulness rests in revealing the dangerously superficial attitude intended to purposely select entire groups, thus supporting the production of a suspect community that generates violent outcomes. This ‘guess who*’* game ends with similar results, with similar types of ‘bodies’ being stopped and searched more thoroughly than others, and it has not changed with technological advancement. As surveillance has been digitized and bodies are scanned via algorithms, it is now evident that some bodies are still more out of place at the border than others (Aas, 2011). This is because facial recognition technology is designed based on discriminatory principles and is tested on predominantly white men (Buolamwini & Gebru, 2018; Harwell, 2019), reproducing the same selective inclusivity/exclusivity that was previously produced manually.

Rather than acknowledging this as a technological problem to address, this discrimination is reinforced further, through manual intervention by immigration officers’ targeting suspect bodies at the border. Stumpf (2006, p. 396) argues that immigration officers are “gatekeepers of membership”, able to define and redefine the conditions of social exclusion because of the merging of criminal law with migration policy, termed as crimmigration. Through this investigative process, Villegas (2015, p. 2357) argues, “practices of humiliation and intimidation are designed to reveal ‘untruths.’” Along the same lines, Friedman (2010, p. 173) refers to the immigration officer as the “arbiter of truth,” implying their self-assigned superior moral status. The “chivalrous spirit” of embracing a moral mission of security and protection (Pratt & Thompson, 2008, pp. 626–627) entrenches this approach, such that the immigration officer goes beyond investigating the truth to producing the truth (van Houtum, 2011). This production of truth favors a particular socio-cultural gendered political view, and hence reflects a bias. Through this process, the border fabricates a truth that fits with the dominant vision of the states of the Global North. Further to this, as pointed out by Marras (2009), state officers—in this case Italian police at the airport—are often unaware of the historical, geo-political, cultural, and linguistic contexts shaping border practices. They are producing truth based on general, anachronistic, and/or uninformed stereotypes. Yet this is not merely random incompetence, and the states’ security narrative provides these officers with impunity (Marmo, 2019).

The theater of paperwork builds on “the fashioning of symbolic objects” (Turner, 1979, p. 465) that has carved out a new discourse of representation of the self. The identity document is the first *item* that is checked, bypassing the human being. Travelers are thus represented by documentary evidence; and so they appear, yet they are invisible as the self (Nield, 2008). Nield (2008) describes how the border as a site of a human being's disappearance is ritualized:

the disaggregation of representation and the subject of representation makes it impossible for the border-crosser to appear – the border becomes a machine of disappearance, and makes a person vanish in plain sight. (p. 138)

This disaggregation between the human and its documentary evidence allows for dehumanized scrutiny, which facilitates the decision to undertake a more personal check.

The theater of the body then takes place when the documentation is judged to be insufficient to reveal the truth. In these cases, holding legal papers of entry is irrelevant (Aas, 2011; Muller, 2004) in influencing the reasons for scrutinizing and/or excluding the suspect communities. Nor does it matter that “some passports are better than others” (Pratt & Thompson, 2008, p. 627), because in some cases even holding a U.S., Canadian, or British passport is not good enough; this othering has a more powerful effect on determinations than the possession of citizenship and other regular documents. “Othered” travelers are not to be trusted, and their travel documentation and oral explanations are therefore often futile – so the theater of the body commences. The immigration system then will focus on the body to locate evidence, based on the spurious explanation that the *body* has to prove the “right to enter” or “right to stay,” even when the individual is entering their home country. At this point of the bordering practice, a distinct and nuanced regime of truth is imposed by the state actor, which transforms the traveling subject into a security concern, privileging the body over speech or paperwork. Yet this is more than a security screening because state power at the border is “enacted through the disciplining of bodies” (Mountz & Hyndman, 2006, p. 451), via the immigration officer's violent performance. The immigration control process, on the one hand, becomes a form of gatekeeping, and on the other, perpetuates the state's hegemonic and possessive power by inscribing such rhetoric and practices onto the traveler's body.

The gendered and racialized body emerges as the subject of interest, and as the object of humiliation and derision. This body appears at the border yet is out of place and must be questioned. So, both the theater of paperwork and the theater of the body serve the purpose of selecting identities, to fulfill wider aims of inclusion/exclusion, and, as discussed in more detail below, to present the performance to audiences.

### As Theatrical Places of Performances (Showing/Doing)

**“**Showing doing” is important in the context of the border because such a performance requires that the audience is uninterested in the details, such as the potentially harmful outcomes for suspect communities (Hillyard, 1993). This audience is far more interested in—and has been well-prepared to receive—a concise, slogan-like securitization message. Enabling the punitive culture of control that is on the rise in the Global North (Garland, 2001) and latching onto “displaced popular anxiety,” borders are being transformed into “screens on which popular fears are projected, and desires for stability, certitude, and security performed” (Walsh, 2015, p. 203). On these screens, patriarchy is also projected, to reassure the audience that hierarchies have not changed, and to target and inject vulnerability into the woman, undermining her agency as a political subject. As pointed out by Andrijasevic (2009), the hegemonic and patriarchal narrative of woman's utility and “decency” as determined through border selection is about constructing gender at the border:

States’ immigration policies need to be understood in relation to the sexual and gendered construction of the nation. Sexuality and gender play a constitutive role in the formation and definition of the nation insofar as the reproduction of nationhood and citizenship remain premised on heterosexuality and heteromasculinity. These denote certain bodies as desirable, and others, in particular, racialized or non-procreative (that is, homosexuals), as being a threat to the nation's survival. (pp. 389–-390)

An all-encompassing ritualized behavior is therefore necessary to have the desired impact, to be interpreted and understood by the audience through the lens we are accustomed to receiving from the state. The border is an ideal space for the state to communicate a message because *any* audience, including but not limited to the traveler, is silenced through various mechanisms employed at the border, including border secrecy. Even when a member of the audience, like a traveler, has a voice, there are compelling reasons not to deviate from the expectation of what can be said: “[t]he setting of a performance, then, communicates the ground-rules for who may speak, what may be said, and what is heard” (Salter, 2008, p. 329). In this ideal theatrical space, visuals, and sounds—as set up and controlled by the state—become of primary importance. It is not just what is displayed but also what is invisible that matters. In fact, the “not-showing … the invisibility, the denial and othering strategy” are all part of the performance, according to Amante (2019, p. 25). The same can be said for sound, as an “enhanced auditory experience” can be created where by new “sonics,” constituted by “wall(s) of noise and quiet manoeuvring,” are projected to the audience in “sound bites” representing threats (Welch, 2012, p. 329).

Border crossing has become a performance in which we all have a part to play, even if we are at home, even if we are not the travelers; we “know” how to act at the border through our experiences, through artistic interpretation, and, most dangerously, through the relentless messages of the state that employ misleading binary statements such as “nothing to hide/nothing to fear.” This, alongside the “way people act and dress, the sequence, form and location of the action” (Amante, 2019, p. 25), contributes to the construction of the narrative that the audience is now *expecting* to receive. The traveler has a clear interest in enacting the rituals and performing the truth at the border, as a theatrical gesture designed to speed up their border crossing, because there is a lot to lose financially (a flight ticket) and emotionally (realization of powerlessness) if they do not comply. The traveler thus enters the space with an expectation of a participatory performance; indeed, audience participation in stage plays has become quite popular (Jordan, 2016). In this performance of security (Amoore & Hall, 2010), where the flow of action is pre-ordained, everyone can be involved, can learn and can act a small part. Often, the audience not only shies away from questioning the performative elements of security, but also endorses the interconnectedness between the site and the security checks enacted within it. In part, this is because the audience believes they themselves represent the “good” traveler who has nothing to hide, and who, at every border passage, passively accepts the necessary symbiosis of site and security at the border, such that one is trusted to be the other: hence, the traveler ends up seeing the site and security as synonymous. And the “good” traveler is rewarded by the state, as pointed out by Pratt and Thompson in their discussion of racial profiling at the border in their study, a Canadian border immigration officer is reported as saying “we don’t want to *bother* the people who are low-risk” (emphasis added) (2008, p. 625). Therefore, the plot unfolds in a cyclical manner with the traveler's active participation, on each occasion confirming the hegemonic stance represented by the border, as pointed out by Salter (2007, p. 59). In Salter's confessionary explanation of how the sovereignty of the border is produced and reinforced, each instance of the audience giving its consent to be scrutinized is an act of border strengthening. Yet this consent is extracted by the entire border machinery, because travelers are trained to approve their own inspection: “travelers are conditioned to confess their history, intentions, and identity, they submit to the examining power of the sovereign” (Salter, 2007, p. 59).

The combined actions of all travelers are thus pivotal to the unfolding of the plot. Any unritualistic demands or any uncertainty displayed by the traveler may delay the crossing or draw unwanted attention. As Browne (2015, p. 135) points out, “the travelling subject is meant to produce herself as trusted and self-regulating.”

Yet, as Browne (2015) argues in her fourth chapter “‘What Did TSA Find in Solange's Fro’?: Security Theater at the Airport,” even with the *correct* ritualistic performance that lacks uncertainty, some bodies stand out as more visible, as more out of place than others, such as the Black woman at the airport in Browne's analysis and the cases presented in the next section.^2^

## Disciplining the Gendered Body—Inscribing State Power onto the Body

The border is a disciplinary site where different forms of power are “inscribed” onto the body (Mountz & Hyndman, 2006). The direct role of state authorities in the *act of inscription* onto the gendered body emerges from the case studies below.

### Strip Searches and Cavity Searches at the Geographical Border

There are many cases of strip searches and intrusive cavity searches conducted at the Mexican–U.S. border without the consent of the woman and without enough suspicion to warrant the officers’ actions. The cases of 18-year-old U.S. citizen Ashley Cervantes (2014), 52-year-old Jane Doe (2012), and 56-year-old Gloria Bustillos (2013) involve similar accounts. These women all held U.S. citizenship, yet, because of their non-white appearance, they were stopped and searched at the border, subjected to a manual cavity search, in some instances by male officers, and forced to undergo a manual medical examination at a nearby hospital, despite the availability of x-ray scanning at the same hospital. In all cases, the women were told they had to cover the hospital bill, which ranged from US$575 to US$5,000 (*Cervantes v. US 2016*; *DOE v. El Paso County Hospital District et al., 2015*; *Bustillos v. El Paso County Hospital Dist. Appellate 2018*). In all three cases no illegal substance was found. The treatment of these women was not just inhuman but plainly illegal (Marmo, 2019). For example, in the case of Ashley Cervantes, she was kept for 7 hrs in the custody of Customs and Border Protection officers without consent and without having her rights read, she was handcuffed to a chair, checked by drug-sniffing dogs, asked to squat so a female officer could visually inspect her, had her request to ring her mother denied, and was then taken to a nearby hospital, where a male physician probed her vagina and anus for drugs as part of an unwarranted body cavity search: “35. Ashley was shocked and humiliated by these exceedingly intrusive searches” (*Cervantes v. US 2016 Lawsuit Case 4:16-cv-00334-CKJ: 8**)*. These three separate incidents demonstrate that the theater of paperwork—holding a U.S. passport to re-enter the United States—is escalated rather quickly into the theater of the body, as the truth must be established by stripping the body and searching inside it.

These are not the only cases of women abused at the southern border of the United States (Johnson, 2012; Staudt, 2008), and they are also not an exception compared to other U.S. borders. At the northern border, there are cases of similar forms of violence, perpetrated by either U.S. or Canadian state agents. For example, in 2016 a woman was detained at the U.S.–Canada border and subjected to an improper strip search and internal body cavity search by immigration officers (Ferriss, 2018; Soloducha, 2016). The following account was reported in the media (Soloducha, 2016, n.p.):

She refused to undress as three men, and no women were in the room, but no other options were given. “It was startling and scary to see those doors go down and having that instant realization I’m trapped in a room with three men.” None of the officers, one who insisted he was the supervisor, would give out their names. “They took my clothes off,” said the woman, her voice breaking with emotion, “…and did an internal cavity search of my body. And it was awful. It was really bad, and I just can’t understand. I just kept saying to them, ‘How could you do this to a woman? This is not right.’” Jason Givens, with U.S. Customs and Border Protection, said a search of this nature would only be conducted if the officer determined beyond all reasonable suspicion that the traveller was carrying contraband. It would also be conducted by a member of the traveller's same sex.

In another case in Canada in 2007, the narrative of being about a wayward officer clearly emerges. In the case *R v. Greenhalgh* (2007*)**,* Canadian border officer Greenhalgh was judged as having abused his power by strip-searching four women and sexually assaulting three of them. The former officer was found guilty of ordering women to remove their clothing and conducting strip searches by himself in inappropriate places such as male toilets. As is evident in this court case, if these incidents are discussed in isolation, they appear as occurrences perpetrated by a particular male officer rather than a systemic issue.

Laughing at and mocking women—which reminds us of Rame's case, as discussed in the prologue—are present in other compelling cases. According to Human Rights Watch, in 1995 a female U.S. border agent manually cavity-searched a woman in the presence of three male agents, who reportedly laughed and joked as they watched (HRW, 1995). In a 2012 case, three Canadian women sued the U.S. immigration border department for “alleg[edly] groping, fondling and mocking” them (Goodyear, 2012). In a 2013 case, while a U.S. female citizen was crying during a very intrusive yet unwarranted search, she was ridiculed and called a “basket case” (Ferriss, 2018).

### Strip Searches and Cavity Searches at Airports

The international airport has become a new borderland of racial and gendered violence. Research on women crossing borders via airports clearly indicates the comparatively high number of African-American women searched at U.S. airports (Browne, 2015; Newsome, 2003). The U.S. General Accounting Office (2000) reported that Black women holding U.S. citizenship were 9 times more likely than their white counterparts to be x-rayed after being searched. Yet they were less than half as likely to be found carrying contraband (Gibeaut, 1999). In the United Kingdom, two government reports from 2011 examining the situation at Gatwick and Heathrow airports revealed a higher number of non-White travelers, whether citizens or holding regular papers, subjected to searches compared to white travelers (Vine, 2011a, 2011b). One of these reports also found that a higher number of women than men were strip-searched (Vine, 2011a). Marras (2009, p. 92) reported the Italian police at an Italian international airport as commenting that “women and children should not be sent around,” infantilizing women as passive and child-like (“sent”) and positioning them as out of place at the airport (“around”). Cases like the one described below evidence the routine abuse of women at airports. Tameika Lovell, a non-white U.S. citizen, was stopped at the New York airport in 2016 and the following occurred:

Inside a secure room, Lovell's litigation asserts, one of the female officers searched Lovell's belongings, presumably for illegal drugs, and asked Lovell if she were using a tampon or sanitary pad. The question upset her, but she replied “no” and complied when told to remove her shoes, lift her arms and spread her legs.

As the other female officer observed, hand on her firearm, the legal document says, the first touched Lovell “from head to toe”, before ordering her to squat. The officer squeezed Lovell's breasts “hard”, and allegedly “placed her right hand into her [Lovell's] pants ‘forcibly’ inserting four gloved fingers into plaintiff's vagina” before parting Lovell's buttocks with her hand “for viewing”. Lovell was left “violated, shocked and afraid” (Ferriss, 2018, n.p.).

In Australia, these searches at the airport also occur but a veil of secrecy is imposed. Only through some rare reports by the Australian National Audit Office (ANAO), is it possible to learn that unlawful strip and internal searches have taken place. For example, in 2017 the ANAO reported that 2,020 personal searches (a combination of strip searches and cavity searches) took place in the 2015–2016 period. Although ANAO analyzed only 69 records out of the full number, the analysis revealed that 10 of those searches were either inappropriate or unauthorized (ANAO 2017; Marmo 2019).

### Onshoring/Offshoring the Gendered Body: The Dynamic Border

The onshore/offshore border detention is a lens that suggests the border is dynamic and that, at any point in time, at any place, the gendered violent border is applicable to the intersectional woman. In Australia's offshore detention facilities, many cases of sexual assault and misconduct have been identified as evidence of the gendered border violence inflicted by state proxies on undocumented women (Australian Senate Inquiry, 2017; Moss, 2015). Reports have included episodes of normalized gendered violence, such as rape and sexual blackmail. For example, detention staff demanded sexual favors from women refugees in return for showers of longer than 2 mins (Caluya, 2019; Cavallaro et al., 2017) and female detainees were offered candies and cigarettes in exchange for sexual favors such as allowing themselves to be seen naked by the state proxy (Moss, 2015, pp. 30–32).

The experiences of undocumented women at the Italian onshore border at the hands of Italian police have been reported in compelling research, albeit as a sideline issue to the more recognized forms of gendered violence (Andrijasevic, 2009; Esposito et al., 2019; Tessitore & Margherita, 2019). While there are no accounts of actual physical abuse of these women by Italian police, it is possible to identify other forms of violence in this space. In Esposito et al. (2019), the reported case studies include stories of women subjected to terrible abuse and violence, yet also reveal the women's strength and agency. For the purpose of this analysis, I extracted these women's observations regarding their treatment at the hands of the Italian police. The Italian police's distrust of non-White undocumented women is a common theme in the literature. Through the lens of the theater, the police can be seen as a theatrical player acting the part of the hegemonic and masculine border, inflicting violence *because* they represent the state. In one case, a woman was verbally abused during a long interview, in which police officers showed their disrespect for her, both in their overall demeanor and by demanding intimate details from her, all the while denying her access to legal representation: “Margarita was held for nine hours by police officers, who treated her disparagingly and asked questions of an intimate nature” (Esposito et al., 2019, pp. 414–415). In a second case, police officers threatened and shouted at the woman while she was crying (Najwa's case study—Esposito et al., 2019, p. 408). Both these women were forced by the Italian police to sign papers without understanding their content because they were in Italian, and without receiving a translation from Italian into their language. The forced paper signing can be understood as an act of infantilization and othering, which is even more disturbing when we consider the human rights treaties to which Italy is a signatory country. For these women, their request for residency was ultimately rejected. Similarly, in a third case, a woman was held in an isolated room by police for 7 hrs without food or water and was provided with no explanation for this treatment (Esposito et al., 2019, pp. 415–416). The police officers referred to her, to her face, in the third person, declaring that they knew what she wanted (to deceive the state in trying to gain residency) and calling her humiliating names of a sexual nature. As in the other cases, this woman was denied legal representation, thus obstructing her access to justice and rendering her more powerless. Given the prevalence and severity of such cases, it is not surprising that many migrant women subjected to exploitation and abuse in Italy do not seek the intervention of the Italian police. As pointed out by Esposito et al. (2019, p. 413), “the threat of being expelled and deported prevented the women from reporting crimes to the police.”

### Mapping Gendered Border Accountability

These cases provide evidence that such violence is perpetrated by state agents or their proxies, supporting the need to map gendered border accountability. The female body at the border becomes liminal, out of place, and it can therefore be trespassed, exploited, humiliated, and devalued while the border is inscribed upon it. Gendered border accountability is required because such cases demonstrate the prevalence of the following: (1) the state's distrust as a default position; (2) the ubiquitous nature of Global North border violence; and (3) the lack of explanation for the selectivity of border violence, which exacerbates further uncertainty and impunity.

First, distrust of women is an all-encompassing premise held by the authorities and is a default position that determines what occurs in domestic cases of family or gendered violence and at the border. This may seem at odds with the fact that the distrust itself is legislated against as an untenable position by state authorities. Breach of the minimum guarantee in criminal proceedings, such as the right to be informed promptly of charges, to access an interpreter, and to have legal assistance are the basic elements of the right to a fair trial and to a fair hearing and the right to the presumption of innocence, as contained in the International Covenant on Civil and Political Rights to which all these Global North countries (Australia, US, UK, Candada) are signatories. Specifically, article 14(3.a) reminds us that the process of determining criminal charges occurs before the actual charges are laid, which is a pivotal legal principle to consider in these cases:

In the determination of any criminal charge against the accused, everyone shall be entitled to the following minimum guarantees, in full equality: (a) To be informed promptly and in detail in a language which the accused understands of the nature and cause of the charge against them.

However, as argued at the beginning of this article, at the border legal rights are temporarily suspended as the narrative of exceptionality and biased assumptions that produce distrust prevail. The distrust is the basis for justifying processes of dehumanization. Such processes have in common the intent to humiliate through various forms, from proactively blocking access to justice to using abusive language and/or behavior that may include unnecessary strip searches and/or cavity searches. Often these forms of abuse are combined.

Second, the ubiquitousness of violence at the border is also an important factor, because it reveals how such treatment of certain travelers is not due to a random wayward officer or to particular socio-cultural stereotypes held by the national authority of an individual country towards certain women. These cases are located in the southern and northern borders of America, in Canada, in Australia, in Italy and in the United Kingdom. In all these locations, women are identified as out of place at the border. The repetition of gendered violence at the borders of the Global North is reminiscent of Dario Fo's theater of situation discussed above. The plot unfolds regardless of the actual individuals. Because the examples provided are just the tip of the iceberg, there is an urgent need for greater state accountability because the problem lies with the system, not with the individual.

Third, the lack of explanation for the selection process may be seen to suggest elements of randomness in the process. The case studies discussed above demonstrate a pattern of acts of violence at the border and that these acts are not rare, and yet they are not collated in to one narrative. Globally, there are many accounts of gendered border violence inflicted by immigration officers and state proxies. While the abusive conduct is systemic and ubiquitous, it is also arbitrary; not all women all the time are subjected to such breaches of their human rights at the hands of the state. However, rather than being reassuring, this causes further uncertainty, as discussed earlier; fluidity and uncertainty produce ambiguity, which cements a vagueness about the application of rules at the border and the reasons for their application. Garland (2001) defines this as the schizophrenic state which appears to be pulling in different directions while advancing its own agenda at all times. The randomness acts as a reminder—to us women—that the body of a woman is never too safe anywhere, and, as with any other form of domestic-gendered violence, women can be *suddenly* disempowered. This too is permanently inscribed on the body of all women everywhere, at all times.

Finally, the examples given above showcase more than just the actualization of gendered and racialized state violence at the border. The fact that these acts are repeated across Global North borders by agents of different national authorities shows their ritualization at a global scale. Reflecting on the mechanisms of ritualization, through the “being”/“doing”/“showing doing” elements introduced in the first part, we can see the theatrical performance in full: “being” the state and holding power over women is the over-arching patriarchal project to maintain; “doing” occurs via the state actions of distrust and body check; the “showing doing” is the message of power the state inscribes onto the woman's body while projecting a message of security to the wider public.

## Conclusion

Rame's monologue “*The Rape*” is embedded in a broader script *Tutta casa, letto e chiesa* (All House, Bed, and Church), implying that, other than at home, in bed and at church, a woman is out of place everywhere.^3^ Hence, she can be attacked, humiliated, and ridiculed, even by state agents and their proxies. By opposing state violence through a theatrical act of dissent, resistance, and empowerment, Rame exposes an uncomfortable and complex truth, a truth that still holds today. We encounter this truth in its unmasked version at the border. The border is thus a theater of truth, where a woman's respect and dignity can be violated by the states of the Global North without much notice and accountability, while the state maintains a rhetoric around the prevention of gendered violence. As pointed out by Freedman (2016):

[i]t is perhaps ironic to note that, whilst many European leaders have recently called for increased action to prevent violence against women, current immigration and asylum policies are pushing some groups of women into situations in which they are at greater risk. (p. 569)

To this set of “situations,” we also need to add the systemic gendered and sexual violence at the border enacted directly by state agents or their proxies. Social sorting at the border ritualizes the formation of alternative and suspect identities, imposed on the traveler through immigration officers’ increasingly coercive powers. This creates a suspension of rights, where a theater of situation can unfold without checks and balances. Despite the many advances in this area, such as the 2019 United Nations Women's Border Management and Gender toolkit, state responsibility for border violence is not considered in the official narrative of the Global North, including in the United Nations toolkit itself.

The gendered border highlights and epitomizes the current state of affairs in the nations of the Global North; despite the rhetoric of policies to fight violence against women, far too often the state betrays its agenda through its treatment of the woman's body. As discussed throughout this article and inspired by the work of Mountz and Hyndman (2006), the border shows us the true nature of the masculine state, masked as security. At the border, the intersectional and contingent nature of gender comes to the fore, and national agendas on the progressive advancement of gender equality are bypassed or suspended. The state empowers itself to establish those exceptions to its own rules, to perform the theater of paperwork and the theater of the body. This represents a spatial arrangement whereby exceptional bodies need to be scrutinized and humiliated as long as the audience is satisfied and applauding.

## Notes

1. According to the most comprehensive and inclusive Italian data (Istat, 2014), 78% of domestic violence victims do not contact any state institution, including police, or seek any other formal support; only 15% of women file a case with the Italian police.

2. See Wilson (2012), on the case of Solange Knowles’ hair searched by Transportation Security Administration officials in 2012 and what happened after that incident.

3. The underlying irony is that women are not safe either at home or in church, but it pleases the masculine state to project such a gendered vision.
